# *Candida albicans*: the current status regarding vaginal infections

**DOI:** 10.1007/s00253-025-13478-2

**Published:** 2025-04-10

**Authors:** Margarida Faustino, Carlos M. H. Ferreira, Ana Margarida Pereira, Ana P. Carvalho

**Affiliations:** 1 Biorbis Unipessoal Lda, Rua Diogo Botelho 1327, 4169 - 005 Porto, Portugal; 2 Universidade Católica Portuguesa, CBQF-Centro de Biotecnologia e Química Fina-Laboratório Associado, Escola Superior de Biotecnologia, Rua Diogo Botelho 1327, 4169 - 005 Porto, Portugal; 3 CBMA (Center of Molecular and Enviromental Biology), Department of Biology, Universidade do Minho, Campus Gualtar, 4710 - 057 Braga, Portugal; 4 IB-S (Institute of Science and Innovation for Bio-Sustainability), Campus de Gualtar, Universidade do Minho, 4710 - 057 Braga, Portugal

**Keywords:** *Candida albicans*, Vaginal infections, Pathogenesis, Prevention strategies, Antifungal resistance, Review

## Abstract

**Abstract:**

Vaginal infections caused by *Candida albicans* are a significant global health concern due to their recurrence and negative impact on quality of life. This review examines the pathogenesis of *C. albicans* infections, emphasizing critical virulence factors such as biofilm formation, adherence, and phenotypic switching. Risk factors include immune system suppression, antibiotic use, and hormonal changes, all of which can lead to fungal overgrowth and infection. Current prevention and/or treatment strategies primarily rely on antifungal therapies, personal hygiene practices, and probiotics. However, challenges like antifungal resistance, recurrence, and limited treatment efficacy highlight the need for innovative approaches. Therefore, emerging methods such as novel antifungal agents, vaccines, and nanotechnology-based delivery systems offer promising advancements to improve infection control. Additionally, the immune system plays a key role in preventing *C. albicans* infections, with both innate and adaptive immunity acting to restrict fungal colonization and growth. Commercially available products, such as antifungal creams, vaginal probiotics, and hygiene solutions, are practical options but often lack long-term efficacy. Persistent challenges, including resistance, patient noncompliance, and restricted access to emerging therapies, hinder comprehensive prevention and treatment efforts. Thus, future research should focus on promoting interdisciplinary approaches, integrating personalized medicine, and enhancing healthcare accessibility. This review intends to present the current state of the art within the abovementioned issues and to enhance the understanding of the multifactorial nature of *C. albicans* infections and advanced prevention strategies, which are essential to reduce the burden of vaginal candidiasis worldwide and improve patient quality of life outcomes.

**Key points:**

• *Candida albicans pathogenesis involves biofilms, adherence, and phenotypic switching.*

• *Vaccines, nanotechnology, and new drugs offer improved prevention and treatment.*

• *Addressing antifungal resistance and patient compliance is key for prevention success.*

**Graphical Abstract:**

**Figure:**
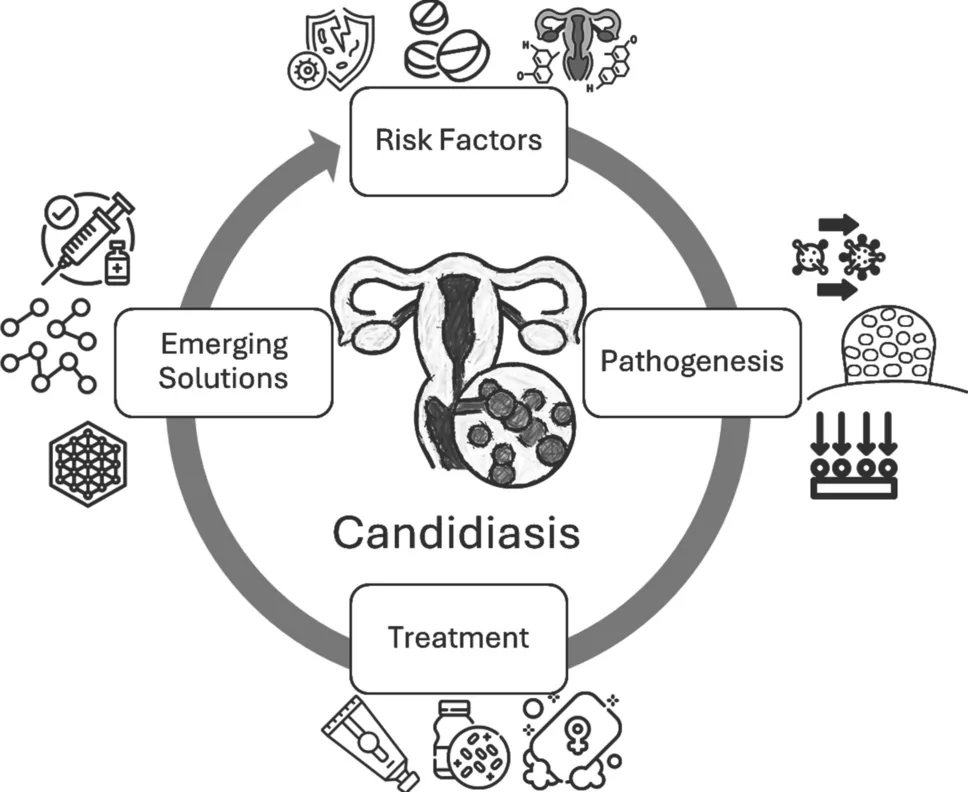

## Introduction

*Candida albicans* is a diploid polymorphic fungus commonly present in several human surfaces such as skin, throat, or vagina mucosa (Parambath et al. 2024). Under certain conditions, such as a weakened immune system, diabetes, pregnancy, or antibiotics therapy, a dysbiosis occurs, and the situation evolves into an infection (Rosati et al. 2020). In the vaginal environment, *Candida* spp. infection is also known as vaginal candidiasis or vulvovaginal candidiasis (VVC). VVC is the second most common vaginal infection (after bacterial vaginosis) and affects 75% of women at least once in their lifetime, although up to 9% of them face recurrent infection episodes, which may surpass 4 per year (recurrent vulvovaginal candidiasis—RVVC) (Rosati et al. 2020). Besides *C. albicans* (the most common species causing invasive diseases), other common species of *Candida* spp. that can overgrow are *Candida glabrata*, *Candida parapsilosis*, *Candida tropicalis*, and *Candida krusei* (Rosati et al. 2020; CDC 2024a).

Although prevention can be achieved through simple strategies such as using cotton underwear and maintaining good hygiene habits, morbidity rates of VVC or RVVC are increasing, as well as the medical costs associated. In fact, *C. albicans* is responsible for more than 150 million mucosal infections per year, leading to healthcare costs of ca. $2 billion in the USA (Richardson 2022). Furthermore, symptoms experienced by women when an infection occurs (vaginal itching, burning, redness, and a white discharge) are often underestimated but limiting to their quality of life (Mayo Clinic 2024). Additionally, despite the pressing need for reliable diagnostic tests and new, safe, and effective treatments and vaccines, research into the mechanisms of fungal vaginal infections is not as advanced as that for other types of diseases. Thus, a systematic overview of the knowledge gathered so far is crucial to understand the current point of situation in terms of available treatments, as well as the new avenues of research that are been drawn for the future. The current review covers the abovementioned issues, focusing on vaginal infections by *Candida albicans*.

### Risk factors for vaginal infections

*C. albicans* infections are influenced by a variety of factors that alter the vaginal microbiota’s normal equilibrium, promoting fungal overgrowth. Recent study continues to provide light on the following critical factors.

- Vaginal microbiome: this dynamic microecosystem plays a crucial role in maintaining vaginal health and preventing infections such as VVC. In healthy women, the vaginal microbiota is typically dominated by *Lactobacillus* sp. which help maintain a low pH environment and produce antimicrobial compounds. However, vaginal dysbiosis, characterized by a decrease in *Lactobacillus* sp. and an increase in diverse microorganisms, can contribute to VVC susceptibility (Ceccarani et al. 2019; Zeise et al. 2021). Studies have shown that women with VVC exhibit significant alterations in their vaginal microbiome compared to healthy individuals. These changes include a reduction in Firmicutes (primarily *Lactobacillus* sp.) and an increase in Actinobacteria, Bacteroidetes, and Proteobacteria (Ceccarani et al. 2019). The dysbiotic state creates a favorable environment for *Candida* sp. overgrowth, particularly *C. albicans*, which is the primary causative agent of VVC (Ceccarani et al. 2019; Valeriano et al. 2024). Furthermore, the formation of polymicrobial associations between *Candida* sp. and other opportunistic microorganisms has been observed in VVC cases, potentially contributing to increased virulence and treatment resistance (Zeise et al. 2021; Li et al. 2022). Understanding the complex interplay between the vaginal microbiome and *Candida* sp. is crucial for developing more effective prevention and treatment strategies for VVC (MacAlpine et al. 2021; Zeise et al. 2021).
- Antibiotic use: antibiotics with a broad spectrum remain a major risk factor for vaginal *C. albicans* infections. Antibiotics can reduce protective vaginal flora, particularly *Lactobacillus* species, which help by producing antimicrobials such as lactic acid. This microbial imbalance enables *C. albicans* to overgrow and invade the vaginal mucosa. *Lactobacillus* sp. depletion lowers competitive exclusion and fungal colonization resistance (Pirotta and Garland 2005; Achkar and Fries 2010; Chee et al. 2020). Antibiotic-induced dysbiosis can increase the recurrence of infections and treatment resistance.
- Hormonal changes: hormonal oscillations, particularly estrogen, affect the vaginal environment. Elevated estrogen levels, which occur during pregnancy, hormonal contraception use, or hormone replacement treatment, stimulate glycogen deposition in the vaginal epithelium, providing a perfect substrate for *C. albicans* development. Estrogen also influences the immunological response, reducing local immune defenses in the vaginal mucosa and generating an environment conducive to fungus persistence (Sobel 2007). This implies that hormonal regulatory mechanisms play an important role in determining susceptibility to infections.
- Immunocompromised states: immunosuppression caused by illnesses such as human immunodeficiency virus (HIV) infection, diabetes mellitus, or the use of immunosuppressive medications dramatically increases the risk of vaginal infections. A compromised immune system reduces the body’s ability to mount effective defenses against fungal infection (Patil et al. 2018). Patients undergoing chemotherapy or those suffering from chronic illnesses frequently encounter recurring vaginal infections because of weakened cell-mediated immunity. Recent research has focused on the involvement of neutrophils and T-cells in vaginal homeostasis and how their failure leads to increased fungal colonization (Köhler et al. 2017). Diabetes and unregulated blood sugar levels also increase the likelihood of recurrent vulvovaginal candidiasis in individuals because increased glucose levels offer a plentiful energy supply to *C. albicans*, boosting its growth and biofilm formation capabilities. Additionally, high blood sugar inhibits the body’s natural defense system, decreasing neutrophils’ effectiveness in fighting off fungal infections (Goderidze et al. 2022).
- Lifestyle factors and hygiene practices: Certain lifestyle behaviors and hygiene habits, such as wearing tight and non-breathable clothing, not practicing good hygiene, and excessive douching, can disrupt the natural balance of vaginal flora, making individuals more prone to *C. albicans* infections. Recent research emphasizes the importance of educational programs on correct hygiene practices to lower infection risks, particularly in women prone to recurring infections (Ventolini and Baggish 2006).

### Pathogenesis of *Candida albicans*

The virulence of a microbial species arises from the interaction between the pathogen and the host rather than being an inherent trait of the microorganism itself. Unlike primary pathogens, which can cause disease without needing a compromised host, opportunistic or facultative pathogens, like *Candida* spp., typically cause infections only in hosts with predisposing vulnerabilities.

**Fig. 1:**
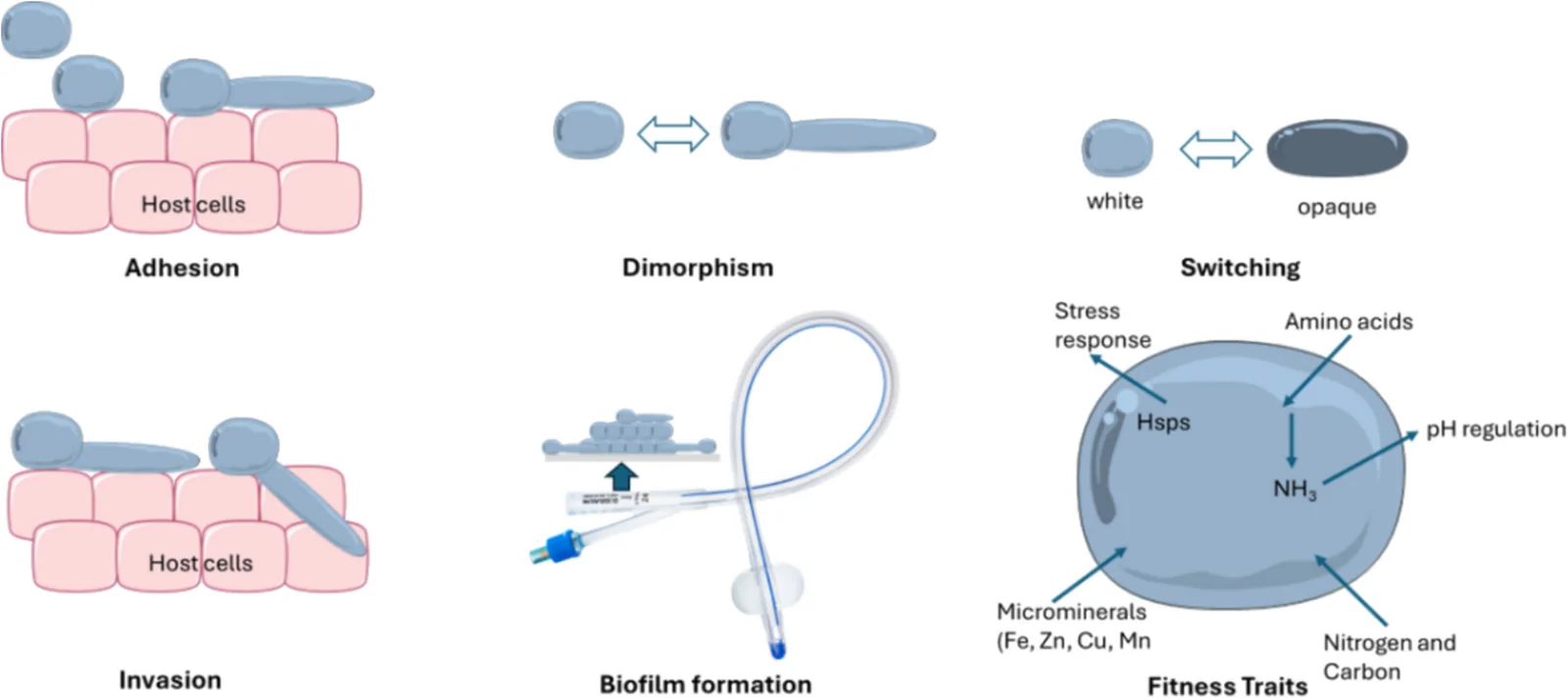
A summary of key pathogenic mechanisms utilized by *Candida albicans*. Adapted from Mayer et al. (2013). The figure was partly generated using Servier Medical Art, provided by Servier, licensed under a Creative Commons Attribution 3.0 unported license

Environmental factors play a crucial role in shaping how pathogens evade or overcome the host’s natural defenses. Virulence is not a static property; it can vary, increasing, decreasing, or potentially returning based on the conditions present (Méthot and Alizon 2014). *C. albicans* has the ability to transform from commensal to pathogenic due to its ability to adhesion, biofilm growth, hydrolytic enzyme release, morphological change, and metabolic adaptability (Fig. 1, Mayer et al. (2013)). In addition, it rapidly adapts to the host environment and infects people with predisposing factors such as antibiotic treatment, malignancy, or weakened immune systems (Tsui et al. 2016; Ciurea et al. 2020).

*C. albicans* is a polymorphic fungus capable of existing as a yeast, pseudo hyphae, or true hyphae. This dimorphism is central to its pathogenicity. In its yeast form, *C. albicans* is adapted for commensal growth on mucosal surfaces. Upon encountering favorable conditions, such as tissue damage or changes in the local environment, *C. albicans* can transition to a hyphal form, which is more invasive. Hyphae penetrate host tissues, facilitating tissue invasion and destruction (Mayer et al. 2013; Chow et al. 2021).

### Mechanisms of infection

There has been a fascinating and extensive range of research into the modes of infection of *C. albicans*. These can be considered as a series of stages, as the organism’s fixation onto the host’s tissues is followed by an evasion from its immune response. In the yeast phase, *C. albicans* resides in the gastrointestinal mucosa, while in the filamentous stage, it penetrates deep into the host tissues to bypass the immune reaction. This transition from yeast to filamentous and vice versa is influenced by favorable environmental conditions, such as tissue damage or changes in temperature, pH, and oxygen levels (Nielsen et al. 2021). The ability of *C. albicans* to stick on epithelial cells is further enhanced by the agglutinin-like sequence (ALS) proteins, which play a fundamental role in the process of infection establishment (Cangui-Panchi et al. 2023).

*C. albicans* initiates infection through a well-orchestrated sequence of events. The first step is adhesion to host tissues, primarily through the activity of ALS proteins. ALS proteins were identified as the first group of factors that mediate adhesion of the fungus to its host cells, by binding to the host cell surface and facilitating colonization of the mucosa. The *C. albicans* fungus contains many ALS proteins, with structural diversities which allow for successful colonization and adaptation to different environmental niches of the host or an indwelling device (Mayer et al. 2013; Lopes and Lionakis 2022).

Once adhered, *C. albicans* invades the host tissues by means of two principal mechanisms: induced endocytosis and active penetration of the host tissues. In induced endocytosis, the fungus is taken up by epithelial cells using a mechanism akin to bacterial intrusion whereby invasins bind to cellular targets and compel their internalization (Wächtler et al. 2012; Sheppard and Filler 2015; Maza et al. 2017; Lachat et al. 2022). In active penetration, *C. albicans* hyphal cells injure epithelial through the secretion of hydrolytic enzymes (mainly aspartyl proteases), which assist in the dissolution of extracellular matrix allowing the invasion into deeper tissues (Wächtler et al. 2012; Mayer et al. 2013).

A critical factor in the persistence of *C. albicans* infections is its capability to develop biofilms. Biofilms suppress fungal exposure to antifungal interventions and immune mechanisms by providing a coating around the fungus. Cells inside biofilms have different patterns of gene expression and metabolite production than their planktonic counterparts, heightening their tolerance to environmental challenges. This ability to develop biofilms is also related with chronic and recurrent infections, as it enables the organism to persist even despite treatment (Nobile and Johnson 2015; Malinovská et al. 2023).

The virulence of *C. albicans* is also potentiated by its ability to produce many hydrolytic enzymes, such as secreted aspartyl proteinases (SAPs) and phospholipases, that are very important in tissue penetration and immune escape. SAPs breakdown host proteins and biofilm structures, assisting the fungus in deeper invasion of the vaginal epithelium (Galocha et al. 2019; Bras et al. 2024). Phospholipases break down the membranes of host cells, hence encouraging them to burst and allowing the fungus to invaginate and spread into the surrounding tissues (Mayer et al. 2013; D’Enfert et al. 2021; Lopes and Lionakis 2022).

Finally, candidalysin is an important virulence factor. Candidalysin is a peptide toxin produced by *C. albicans*, specially by its pathogenic hyphal form and functions by causing direct epithelial damage and provoking inflammatory signaling (Ho et al. 2020). Ho et al. (2019) found that candidalysin activates the epidermal growth factor receptor (EGFR), triggering mitogen-activated protein kinase (MAPK) signaling. In addition, candidalysin stimulates the release of alarmins and antimicrobial peptides, further underpinning host immune responses (Ho et al. 2020).

These adhesion, invasion, biofilm formation, and enzyme and toxin production processes show how complex *C. albicans* mechanisms are in acquiring lesions, maintaining them and reinfecting the host.

### Host–pathogen interactions

The interaction between *C. albicans* and the immune system of the host is a complex and dynamic system that encompasses innate and adaptive immune components. The innate immune system is predominantly effective in the initial stages of recognition and control of *C. albicans* colonization and infection, through the actions of neutrophils, macrophages, and dendritic cells (DC). These immune cells express a few pattern recognition receptors (PRRs) such as Toll-like receptors (TLRs), C-type lectin receptors (CLRs), and nucleotide-binding oligomerization domain (NOD)-like receptors (NLRs) that recognize and respond to the fungal cell wall and promote intracellular processes such as ingestion of the fungi and secretion of inflammatory cytokines to curb the fungal propagation (Dühring et al. 2015; Zheng et al. 2015; Lionakis et al. 2023). However, *C. albicans* has evolved various immune evasion strategies that allow it to persist within the host, often leading to chronic or recurrent infections. One such mechanism involves masking key elements of its cell wall, particularly β-glucan, during hyphal growth. β-Glucan is a potent activator of immune responses via PRRs like Dectin- 1, but by camouflaging it, *C. albicans* reduces its visibility to immune cells, thereby preventing robust immune activation (Mata-Martínez et al. 2022). In addition, the biofilm formed by *C. albicans* not only enhances its survival in hostile environments but also provides a physical shield that impedes immune cell activity, further complicating the elimination of the pathogen (Hernández-Chávez et al. 2017; Garcia-Rubio et al. 2019).

In addition to innate immunity, the adaptive immune system, particularly Th17 cells (subsets of T cells), plays a crucial role in controlling *C. albicans* infections. Th17 cells produce IL- 17, a cytokine that recruits neutrophils and enhances fungal clearance. However, *C. albicans* can reduce the Th17 response in chronic infections, promoting persistent infections by reducing immune control (Garcia-Rubio et al. 2019).

### Role of the immune system

When the delicate balance between *C. albicans* and the immune regulation is disrupted, it can lead to an overgrowth of *C. albicans*, resulting in infection. Understanding the mechanisms by which the immune system regulates this balance is critical for developing strategies to prevent vaginal infections. A deeper knowledge of how both innate and adaptive immune defenses function in this context can provide valuable insights into therapeutic interventions and preventive measures against *C. albicans* infections.

#### *Innate immune responses*

The innate immune response acts as the first line of defense against *C. albicans* in the vaginal mucosa. In the context of vaginal infections, four key components play dominant roles: epithelial cells, PRRs, cytokines, and the NLRP3 inflammasome.

- Epithelial cells: vaginal epithelial cells (VECs) can distinguish between the commensal and pathogenic forms of *C. albicans*, responding differently based on fungal morphotype and load (Gaziano et al. 2023). Although VECs exhibit some antifungal activity, their effectiveness is lower compared to oral epithelial cells, for example (Barousse et al. 2005). Another factor influencing the protective efficacy of VECs is the vaginal environment, particularly pH levels. More acidic environments have been shown to reduce the antifungal activity of VECs (Barousse et al. 2005). On the other hand, certain interventions can enhance the efficacy of VECs against *C. albicans*. For instance, specific probiotic strains have been shown to strengthen the barrier function of VECs and improve their ability to distinguish between commensal and pathogenic forms of *C. albicans* (Dong et al. 2023). Additionally, topical estrogen application can promote VEC proliferation and maturation, potentially reinforcing the vaginal epithelial barrier against *C. albicans* (Sibeko et al. 2024).
- Pattern recognition receptors (PRRs): innate immune cells recognize *C. albicans* through various PRRs, including CLRs like Dectin- 1 and Dectin- 2 (Richardson and Moyes 2015). While these PRRs are critical for detecting *C. albicans*, they also recognize other fungi and microorganisms, leading to broader immune responses. This broad recognition capability allows these receptors to play important roles in defending against multiple fungal pathogens, though it means their activation is not specific to *C. albicans* alone (Robinson et al. 2009; Saijo and Iwakura 2011). Furthermore, *C. albicans* has evolved sophisticated strategies to evade or modulate PRR recognition, potentially limiting the effectiveness of the innate immune response. One such example involves shielding of β− 1,3-glucan, a key pathogen-associated molecular pattern (PAMP), with an outer mannan layer. This “hiding” strategy reduces the reactivity of Dectin- 1, against *C. albicans* yeast cells (Hernández-Chávez et al. 2017).
- NLRP3 inflammasome: the NLR family pyrin domain-containing 3 (NLRP3) is a multiprotein complex comprising an amino-terminal pyrin domain (PYD), a carboxy-terminal leucine-rich repeat domain (LRR domain), and a central NACHT domain thus named to its presence in the neuronal apoptosis inhibitor protein (NAIP), the major histocompatibility complex class II transcription activator (CIITA), the incompatibility protein locus from the fungus *Podospora anserine* (HET-E), and the mammalian telomerase-associated proteins (TP1) (Koonin and Aravind 2000; Swanson et al. 2019). After host cells membrane damage (namely potassium efflux) by candidalysin or *Candida albicans* recognition via binding of β-glucan to Dectin- 1, NLRP3 inflammasome is activated in macrophages, leading to the activation of caspase- 1 that promotes the formation of the proinflammatory cytokines interleukin- 1β (IL- 1β) and interleukin- 18 (IL- 18) and pyroptosis (Tavares et al. 2015; Rogiers et al. 2019; Swanson et al. 2019; Chen et al. 2023; Xu et al. 2024). In women with VVC and RVVC, the strong inflammation response driven by these cytokines, as a defense mechanism against infection, causes several symptoms such as irregular and thick white vaginal discharge, pain, burning, itching, redness, and swelling in the vulva and/or vagina (Cheng et al. 2024). Roselletti et al. (2017) have reported that the expression of NLRP3 inflammasome is significantly higher in women with VVC when compared to asymptomatic carriers. Furthermore, genetic polymorphisms in the gene encoding NLRP3 have shown to prompt exacerbation of the inflammatory response to *C. albicans* (Bruno et al. 2015), thus emphasizing the role of genetic polymorphisms of the NLRP3 inflammasome in the pathogenesis of VVC and RVVC. NLRP3 inflammasome inhibitors have gained interest as a therapeutic strategy, with several research studies reporting reduced inflammation in several disease models (Zahid et al. 2019; Mezzaroma et al. 2021; Ambrus-Aikelin et al. 2023; Xu et al. 2024; Tantra et al. 2024). A reduction in the inflammatory response in VVC, namely, the decrease of IL- 1β expression levels, has also been shown for mice when intravaginally administered with glyburide, an effective inhibitor of the NLRP3 inflammasome (Bruno et al. 2015). This observation supports the belief that by modulating the inflammasome activity, NLPR3 inflammasome inhibitors are promising therapeutic agents in the management of VVC and RVVC, not only by alleviating the symptoms and discomforts experienced by women with VVC/RVVC but also by reducing the frequency of recurrent infections.
- Cytokine production: Activated epithelial cells release pro-inflammatory cytokines and chemokines that help combat *C. albicans* infections, and the application of specific pro-inflammatory cytokines or their analogs could enhance the local immune response against *C. albicans* (De Bernardis et al. 2015). However, while pro-inflammatory cytokines are essential for recruiting immune cells, excessive inflammation can lead to tissue damage and symptoms associated with vaginal candidiasis (Santoni et al. 2002) Furthermore, an overproduction of cytokines may disrupt the balance between tolerating commensal *C. albicans* and responding to its pathogenic forms. Additionally, *C. albicans* has evolved mechanisms to modulate host cytokine responses, potentially limiting the effectiveness of this immune strategy. These mechanisms include manipulating T cell responses (Swidergall and LeibundGut-Landmann 2022), altering macrophage phenotypes, and producing cytolytic peptide toxins (Zhao et al. 2022).

#### *Adaptive immune responses*

The adaptive immune response, particularly T cell-mediated immunity, is essential for long-term protection against *C. albicans* infections. Here, several particularly important factors subsist; CD4^+^ T helper (Th) cells, DCs, CD8^+^ T cells, and antibody-mediated immunity.

- CD4^+^ Th cells: these, especially the Th1 and Th17 subsets, play a pivotal role in this defense. CD4^+^ T cells are regarded as the primary cell-mediated adaptive immune response against *C. albicans* (Richardson and Moyes 2015). This is evident in patients with acquired immunodeficiency syndrome (AIDS), who, due to a lack of CD4^+^ T cells, exhibit higher susceptibility to *C. albicans* infections (van de Veerdonk and Netea 2010). Th1 cells contribute to antifungal defense by producing IFN-γ, which induces nitric oxide production in macrophages and promotes the generation of *Candida*-specific antibodies (van de Veerdonk and Netea 2010). Meanwhile, the Th17 subset is a double-edged sword regarding VVC control. Th17 cells produce cytokines IL- 17 (among others), which are both beneficial and detrimental in host defense against disseminated candidiasis. In one hand, overproduction of IL- 17 A is linked to inflammatory and neutrophil recruiting, which in turn lead to tissue damage and symptomatic VVC (Bagri et al. 2022). On the other hand, studies show that mice deficient in Th17 responses are highly susceptible to oropharyngeal candidiasis, and IL- 17 receptor knockout mice display increased vulnerability to systemic *Candida* infections compared to wild-type mice (Pathakumari et al. 2024). Together, these findings underscore the vital roles of Th1 and Th17 responses in protecting against *C. albicans*.
- Antibody-mediated immunity: In addition to T cells, B cells and antibodies also contribute to protection, albeit to a lesser extent (Santoni et al. 2002). For example, authors have demonstrated that when B cells were transferred to naive rats, these animals showed fewer *Candida* sp. colony-forming units compared to controls. However, the rate of fungal clearance was slower than that observed in animals receiving immune T cells (Santoni et al. 2002).
- Dendritic cells: although not part of the adaptive system, they play a pivotal role in bridging innate and adaptive immunity by driving specific T cell responses against *C. albicans*. Different subsets of DCs are specialized in promoting distinct immune pathways. For instance, Langerhans cells, a type of DC found in epithelial tissues, are key in eliciting Th17 responses, which are critical for antifungal defense. On the other hand, Langerin^+^ dermal DC stimulate Th1 responses and cytotoxic T lymphocyte activity, thereby contributing to a broader immune response that includes both antifungal and cytotoxic mechanisms (Richardson and Moyes 2015).
- CD8 + T cells: Although CD8^+^ T cells are less effective than CD4^+^ T cells in controlling *C. albicans* infections, they still play a contributory role in fungal clearance. By recognizing and targeting infected cells, CD8^+^ T cells add an additional layer of defense to the immune response. Their cytotoxic activity, although not as central as the helper functions of CD4^+^ T cells, supports overall fungal elimination, particularly in disseminated infections where robust immune engagement is required (Santoni et al. 2002).

#### *The right balance*

Maintaining a balance between immune activation and tolerance is crucial for preventing unnecessary inflammation while ensuring effective responses to *C. albicans* infections. Several factors are key to prevent infection in this context:

- Commensal vs. pathogenic forms: The immune system must accurately distinguish between commensal and pathogenic forms of *C. albicans* to prevent harmful immune overactivation. In its commensal state, *C. albicans* exists as a harmless yeast, coexisting with the host without causing tissue damage. However, under certain conditions, such as immunosuppression or environmental changes, it can switch into its pathogenic hyphal form, triggering immune activation (Gaziano et al. 2023). This distinction is primarily achieved through pattern recognition receptors (PRRs), such as C-type lectin receptors (CLRs) like Dectin- 1 and Dectin- 2, which sense fungal cell wall components and distinguish morphotypes. The epithelial cells and immune cells work together to detect fungal load, morphology, and other danger signals, ensuring that only pathogenic forms elicit a significant immune response.
- Immune tolerance: in healthy women, VECs exhibit a remarkable ability to tolerate *C. albicans* colonization without initiating an excessive inflammatory response (Gaziano et al. 2023). This immune tolerance is essential for maintaining a symbiotic relationship with *C. albicans*, preventing unnecessary tissue damage caused by immune overactivation. VECs achieve this through tightly regulated signaling pathways that modulate the production of pro-inflammatory cytokines and antimicrobial peptides in response to commensal fungal forms (Gaziano et al. 2023).

Together, the delicate balance between immune activation and tolerance relies on a dynamic interplay of host recognition systems, fungal behavior, and environmental factors. Understanding how this balance is maintained, and what factors disrupt it, is key to identifying strategies for preventing and managing vaginal candidiasis.

### Current prevention strategies

*C. albicans* infections are practically inevitable, as these opportunistic fungal pathogens are ubiquitous commensals of humans (Shao et al. 2022), while asymptomatically colonizing the skin and mucosal surfaces of the oral cavity and the gastrointestinal and reproductive tracts of healthy individuals (Achkar and Fries 2010; Alonso-Monge et al. 2021; Lemberg et al. 2022). *C. albicans* can switch from harmless to pathogenic under certain conditions such as microbiota dysbiosis, impaired immune system, damage to the skin or mucosal barriers, changes in environmental pH or nutrient availability, and virulence gene expression by the fungus (Fidel 1999; Talapko et al. 2021; Jacobsen 2023).

**Fig. 2:**
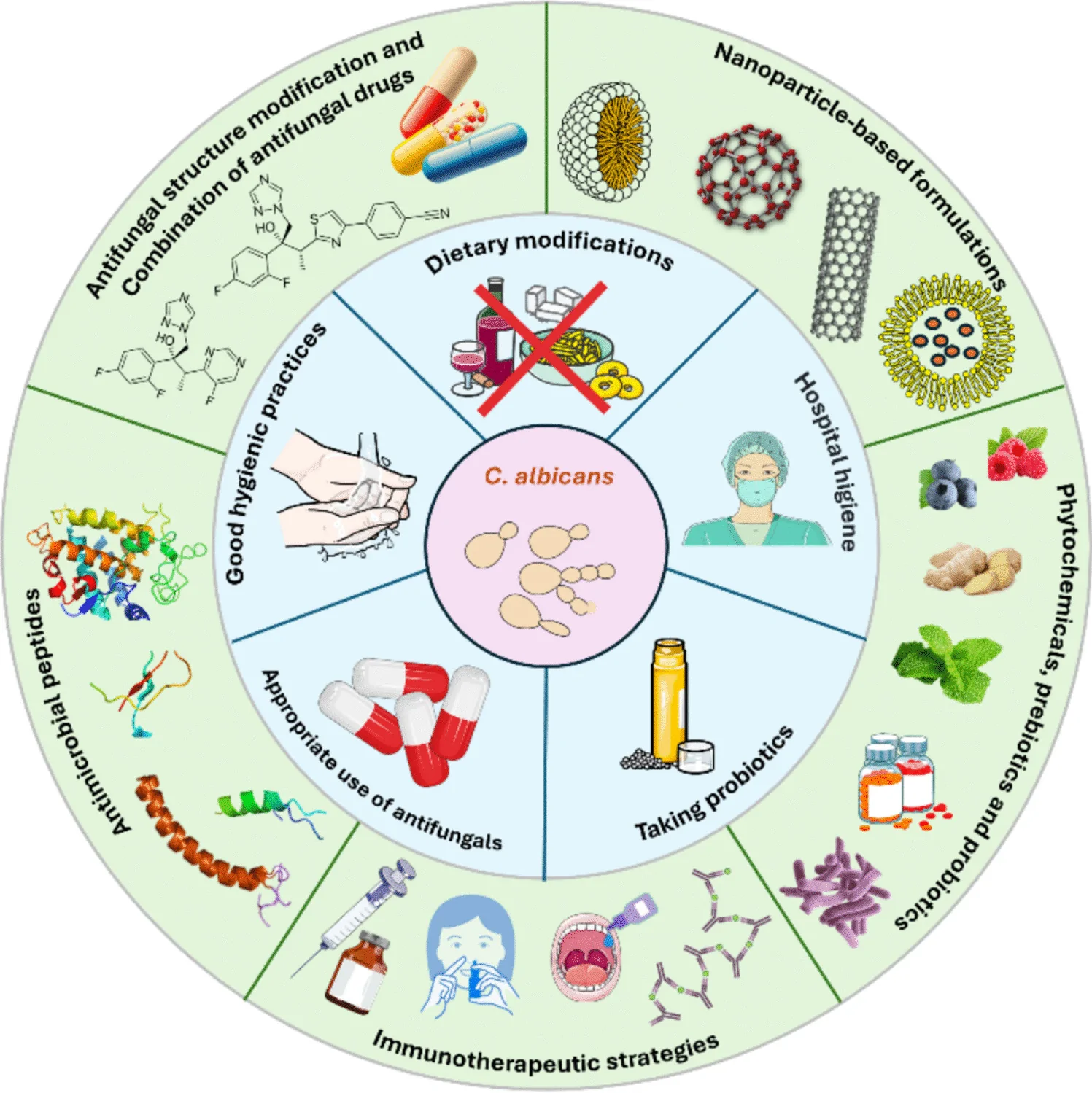
Conventional (inner blue circle) and emerging (outer green circle) strategies to prevent *C. albicans* infections

Several approaches can be used to prevent *C. albicans* infections, namely (Fig. 2), maintaining good hygienic practices (hands and bathing with suitable soap regularly, keeping the external genitalia clean and dry), wearing cotton and breathable underwear, practicing safe sex, avoiding the consumption of dispensable antibiotics, and managing diabetes (CDC 2024b; Martins et al. 2014), as patients with this disease are more susceptible to yeast infections. Taking probiotics can also be beneficial for managing *Candida* spp. infections. Indeed, different *Lactobacillus* and *Bifidobacterium* genera species have been widely used for many decades as probiotics, as they provide a health benefit to the host by inhibiting *Candida* spp. growth (Chew et al. 2015; Matsubara et al. 2016; Hernãndez-Bautista et al. 2020). Dietary modifications could also be beneficial to prevent *C. albicans* overgrowth. Accordingly, alcoholic beverages, bread, and fermented products should be avoided, as these foods already contain a high quantity of fungi or yeast. A low-sugar diet (deprived of refined sugars, sucrose, honey, corn syrup, and fruit juice) should also be adopted since *C. albicans* uses sugar as carbon source to grow. In addition, allergenic foods such as milk, dairy products, eggs, wheat, or peanuts should be removed from the diet to prevent the weakening of the immune system and subsequent growth of the pathogen (Martins et al. 2014).

Nosocomial *Candida* spp. infections can also be prevented by adopting standard precaution methodologies. To avoid the dissemination of these hospital-acquired fungal infections, it is crucial for healthcare workers to wear personal protective equipment (gowns, gloves, masks, eye and face shields, shoe covers, head covers…), frequently wash their hands with soap (plain or with antiseptic), and rub them with an alcohol-based sanitizer (CDC 2024c). Noncritical environmental surfaces must be cleaned and disinfected, as well as medical equipment, instruments, and devices that require thorough decontamination before being used on a different patient. Indwelling devices (such as enteral feeding tubes, endotracheal tubes, arterial and central venous and catheters, urinary catheters and drains) should be used only, when necessary, inserted after disinfection of the skin and removed as early as possible. Additionally, high risk patients should be given appropriate antifungal prophylaxis, infected patients should be kept apart from other patients (either in single room isolation or cohosting), and the use of unnecessary antibiotics (particularly broad-spectrum antibiotics) on patients must be avoided (Ture and Alp 2018; CDC 2024c). However, *C. albicans* infections can still occur and become threatening if not treated properly.

Current available pharmacotherapeutic approaches for *C. albicans* treatment rely only on four classes of US Food and Drug Administration (FDA) approved antifungal drugs: polyenes, azoles, echinocandins, and 5-flucytosine (Salazar et al. 2020; Wall and Lopez-Ribot 2020). The following table (Table 1) summarizes the main classes of antifungal drugs, some examples and mechanisms of action.

**Table 1:** Overview of antifungal drug classes and their mechanisms of action

Class of antifungal drugs	Examples	Mechanism of action	References
Polyenes	Amphotericin B, nystatin	Penetrate fungal cell membrane and bind to ergosterol, forming pores that cause leakage of cellular content, leading to cell death	(de Oliveira Santos et al. 2018; Salazar et al. 2020; Wall and Lopez-Ribot 2020)
Azoles	Fluconazole, ketoconazole, clotrimazole, butoconazole, miconazole, econazole, itraconazole	Inhibit ergosterol biosynthesis, reducing membrane integrity, resulting in cell lysis and death	(Maertens 2004; de Oliveira Santos et al. 2018; Scorzoni et al. 2021)
Echinocandins	Caspofungin, anidulafungin, micafungin	Inhibit β-(1,3)-D-glucan synthase, blocking β-(1,3)-D-glucan synthesis, a key fungal cell wall component, causing cell lysis and death	Death (Salazar et al. 2020; Mroczyńska and Brillowska-Dąbrowska 2020; Szymański et al. 2022)
5-Flucytosine	5-Fluorocytosine	Enters fungal cells via cytosine permeases and is converted to 5-fluorouracil, inhibiting RNA and DNA synthesis, leading to cell death	(Salazar et al. 2020; Wall and Lopez-Ribot 2020)

Unfortunately, none of these conventional antifungal drugs has the optimum profile, as all of them are associated with some limitations

The increased use of antifungal agents over the years resulted in the emergence of antifungal resistance, namely, among *Candida* species. Resistance phenomenon in yeast can be intrinsic (primary) or acquired (secondary). Intrinsic resistance occurs prior to antifungal exposure because of their fundamental structure that hinders the binding to its drug target (de Oliveira Santos et al. 2018; Czajka et al. 2023). This is the case, for example, of the fluconazole-resistant *C. krusei* or the less echinocandins-susceptible *C. parapsilosis* (Salazar et al. 2020; Murphy and Bicanic 2021; Czajka et al. 2023). Differently, acquired resistance is an evolutionary response to antifungal selective pressure, leading to genetic mutations such as modification or elimination of antifungal drug target and protein channels required for the uptake of the antifungals by the yeasts, modification of the composition of the fungal cell membrane, or the overexpression of efflux pumps critical for the expelling of the drugs from yeasts (de Oliveira Santos et al. 2018; Wall and Lopez-Ribot 2020; Murphy and Bicanic 2021; Czajka et al. 2023). Examples of this type of resistance mechanisms have been observed in *Candida* species such as *C. albicans*, *C. glabrata*, and *C. auris*, in response to azoles, echinocandins, polyenes, and 5-flucytosine (Salazar et al. 2020; Murphy and Bicanic 2021; Czajka et al. 2023). Additionally, phenotypic alterations often decrease fungal susceptibility to antifungals. Indeed, *Candida* species are known to form biofilms, thus living within a self-produced polymeric matrix that acts as an additional barrier that limits the distribution of the antifungal drugs (Murphy and Bicanic 2021; Kaur and Nobile 2023; Czajka et al. 2023).

Other limitations of the current conventional antifungal drugs regard their pharmacokinetics and cytotoxicity (Ostrosky-Zeichner et al. 2010; de Oliveira Santos et al. 2018; Wall and Lopez-Ribot 2020; Roy et al. 2023; Czajka et al. 2023). For example, within the azole family, not all antifungals can be used as systemic medication. Due to their high toxicity and low bioavailability, imidazole-based azoles (such as clotrimazole, miconazole, econazole butoconazole, or ketoconazole) can only be formulated for superficial fungal infections (Ostrosky-Zeichner et al. 2010). While exhibiting low toxicity, echinocandins have the great disadvantage exhibiting poor oral bioavailability due to their larger molecular weight (being available only in intravenous formulations), and amphotericin-B, although extremely efficient, has been reported to be responsible for nephrotoxicity, electrolyte irregularities, and anaphylaxis events, besides being practically insoluble in water (Wall and Lopez-Ribot 2020; Murphy and Bicanic 2021). In addition, recurrent usage of many antifungal agents is limited due to their nephrotoxicity and hepatotoxicity (de Oliveira Santos et al. 2018; Murphy and Bicanic 2021).

To overcome all the abovementioned challenges related to the current antifungal pharmacotherapies, novel approaches must be exploited.

### Innovative and emerging prevention methods

To minimize the different limitations associated with conventional antifungal drugs, different pharmacological strategies have been exploited (Fig. 2). Structure modifications and new formulations of the available antifungals have been explored for the development of new antifungal compounds. New modified triazoles (e.g., ravuconazole, albaconazole, and isavuconazole) and tetrazoles (oteseconazole) have been shown to have in vitro activity against *Candida* species such as *C. albicans*, *C. glabrata*, *C. parapsilosis*, *C. krusei*, and *C. auris* strains while exhibiting low cytotoxicity. Specifically, voriconazole and posaconazole, two second generation triazoles with broad-spectrum activity, have been reported to have superior antifungal activity against most *Candida* species (de Oliveira Santos et al. 2018; Scorzoni et al. 2021). Similarly, alterations in the molecular structure of echinocandins give rise to new antifungal molecules (such as rezafungin) with reduced probability to elicit fungal resistance, increased antifungal potency against *Candida* species, and better pharmacokinetic profile (Scorzoni et al. 2021; Jospe-Kaufman et al. 2024). Liposomal formulations of amphotericin B were also developed aiming to surpass its limitations. The incorporation of amphotericin B into liposomes, lipid complexes, or colloidal dispersions allowed the development of new antifungal targeted therapies while reducing its toxicity and enhancing its pharmacokinetics (Torrado et al. 2008; Salazar et al. 2020). Three intravenous infusions of these liposomal formulations (Ambisome®, Abelcet®, and Amphotec®) are already commercially available (Torrado et al. 2008), and an oral formulation (CAmB/MAT2203) is under clinical trials for the treatment of invasive candidiasis (Jenssen et al. 2006).

Exploitation of antimicrobial peptides is a promising alternative to antifungal drugs. Ubiquitous in nature, these short, amphipathic, positively charge molecules are acknowledged to electrostatically interact with the membrane, leading to membrane permeabilization and consequent inhibition of nucleic and ribonucleic acid synthesis, protein and cell wall synthesis, and enzymatic activity (Jenssen et al. 2006). Within these antimicrobial peptides, several have been reported to have antifungal activity, namely, anti-*Candida* activity in both planktonic and sessile physiological states (Fernández de Ullivarri et al. 2020; Perez-Rodriguez et al. 2022). These include human defensins- 1, − 2, and − 3 (HBD- 1, HBD- 2, and HBD- 3, respectively), found to be active against *C. albicans*, human lactoferrin (hLF) that has been reported to display activity against *C. albicans and C. krusei*, bovine cateslytin (bCAT), found to be active against *C. albicans*, *C. glabrata*, and *C. tropicalis*, human histatin- 5 (Hst 5) that has revealed activity against *C. albicans* and to inhibit its biofilm formation, LL- 37, that holds antifungal activity against planktonic and sessile *C. albicans* and *C. auris*, and also lysozyme that has been demonstrated to be active against several *Candida* species (de Oliveira Santos et al. 2018; Perez-Rodriguez et al. 2022).

Another strategy to enhance the therapeutic efficiency of the existing antifungal drugs consists in their combination with each other or with new molecules, as it can overcome their toxicity hurdle by reducing the individual doses of each agent, besides reducing the risk of antimicrobial resistance emergence due to the implication of multiple drug targets simultaneously (Scorzoni et al. 2021). The synergetic effect of the combination of 5-flucytosine and amphotericin B has been reported to have an improved effect to treat invasive candidiasis (Scorzoni et al. 2021). Similarly, the combination of azoles and echinocandins (e.g., fluconazole + micafungin, voriconazole + micafungin, posaconazole + caspofungin), the combination of polyenes and echinocandins (e.g., amphotericin B + caspofungin, amphotericin B + anidulafungin, amphotericin + micafungin), and the combination of polyenes and azoles (e.g., amphotericin + posaconazole) have shown that their synergistic antifungal effect against *Candida* spp. infections and biofilms was enhanced when compared to their individual activities (de Oliveira Santos et al. 2018; Scorzoni et al. 2021). Moreover, the combination of lactofungin, a lactoferrin-derived antifungal peptide, with amphotericin B demonstrated the potential of this new compound to be efficient used in *anti-Candida* therapies while using a low dosage and, thus, prompting a reduction on amphotericin B cytotoxicity (Fernandes et al. 2020).

As stated before, nanoparticles such as liposomes have been exploited as antifungal delivery systems to fight candidiasis fungal infections. Different nano systems have been assessed for alternative antifungal therapies such as solid lipid nanoparticles, nanostructured lipid carriers, polymers, and metals with innate antimicrobial properties (e.g., zinc, gold, and silver). Their small size and high surface area-to-volume ratio allows the encapsulation, adsorption or chemical attachment of considerable amounts of antifungal agents, and transportation to specific targets, without being recognized by efflux pump proteins (Salazar et al. 2020; Scorzoni et al. 2021). Nanostructured lipid carriers loaded with amphotericin B revealed enhanced antifungal activity when compared with the commercial amphotericin B colloidal system Fungizone®, and solid lipid nanoparticles loaded with fluconazole demonstrated improved activity against *C. albicans*, *C. glabrata*, and *C. parapsilosis* (Scorzoni et al. 2021). Likewise, fluconazole-loaded chitosan nanoparticles exhibited considerable activity against the same *Candida* species, and silver nanoparticles (either alone or conjugated with fluconazole) have also been shown to be highly effective against *Candida* species (Salazar et al. 2020; Scorzoni et al. 2021).

In recent years, immunotherapeutic strategies have been gaining a special interest for the treatment of *Candida* infections. Particularly, great efforts are being made in the development of vaccines to prevent *Candida* infections through stimulation of the immune system to recognize the fungus and consequently attack it by eliciting an immune response. Although no *Candida* sp. vaccines are currently clinically available, two formulations are already in clinical trials (Costa-Barbosa et al. 2023; van de Veerdonk et al. 2010), and many others are being developed and assessed for their application in anti-*Candida* therapies. Several types of vaccines with different routes of administration (injectable, oral, intranasal) are being explored to prevent *Candida* infections (Sahu et al. 2022; Alapan et al. 2024). This immunotherapeutic approach has significantly evolved by taking advantage of genetic editing techniques such as the clustered regularly interspaced short palindromic repeats (CRISPR)-Cas9 system that not only allows the identification of new targets for novel antifungal compounds but also allows the creation of attenuated *Candida* strains that can be used as live vaccines (Uthayakumar et al. 2020). Live attenuated whole-cells are designed to contain a weakened form of the whole fungus that will cause a minor infection which will not evolve to a severe disease but instead will produce a robust long-lasting immune response against subsequent exposure to the pathogen (Sahu et al. 2022; Alapan et al. 2024). This kind of vaccination has already proved to avoid reinfection in animal models after being inoculated with a low virulent *C. albicans* strains (van de Veerdonk et al. 2010; Sahu et al. 2022). However, some concerns regarding this type of vaccines concern their application in immunocompromised patients, who may become seriously ill after inoculation (Sahu et al. 2022). Differently, killed whole cell vaccines use inactivated *Candida* cells (killed by radiation, chemicals, or heat) to stimulate an immune response, thus being safer for patients with a reduced ability to fight infections and diseases (Sahu et al. 2022; Alapan et al. 2024). Indeed, inoculation of inactivated *C. neoformans* conferred immune protection against several fungi pathogens in immunocompetent and immunocompromised animal models (Alapan et al. 2024). Alternative anti-*Candida* vaccines have been designed to use only parts of *Candida* such as proteins, peptides and polysaccharides, or genetic material, such as DNA and RNA, to trigger an immune response (Sahu et al. 2022; Alapan et al. 2024). Recombinant vaccines based of *C. albicans* adhesins (agglutinin-like sequence 1 and 3; Als1p and Als3p) and *C. parapsilosis* secreted aspartic protease 2 (Sap2) protein revealed enhanced immunological protection against fungal infections in animal models (van de Veerdonk et al. 2010; Sahu et al. 2022), and a heat shock protein from *C. albicans* (hsp90-CA) encoding DNA vaccine showed a protective immunological response to vaginal candidiasis in animal models. In addition, conjugate fungal vaccines composed of fungal antigen such as (polysaccharides) covalently linked to carrier proteins have also demonstrated to be a promising alternative, namely, against *Candida* vaginal infections (van de Veerdonk et al. 2010; Sahu et al. 2022).

Non-pharmacological approaches, using phytochemicals, prebiotics, and probiotics, have also been exploited against candidiasis. Several oily formulations of plant extracts have already been topically used as therapeutics for vulvovaginal candidiasis, namely, oils from lemon balm, garlic, fennel, chamomile, ginger, and sage (Picheta et al. 2024). Additionally, molecules such as allicin, curcumin, cannabidiol (CBD), and dill oil have shown to have a beneficial effect on vulvovaginal candidiasis, whereas green tea, cinnamon, garlic, propolis, and ginger revealed *anti-Candida* activity against oral candidiasis (Gharibpour et al. 2021; Picheta et al. 2024). These findings support the idea that plant-based bioactive compounds can be a good option for the treatment of different *Candida* infections. Finally, it is well known that the consumption of probiotics has several benefits for human gut health. Similarly, the use of these “generally recognized as safe” (GRAS) live microorganisms has also been used for the maintenance and improvement of women vaginal microenvironment. When the abundance of lactobacilli within the vaginal tract (mainly *L. gasseri*, *L. jensenii*, and *L. crispatus*) decreases, an increase on the pathogenesis of *Candida* species (namely, *C. albicans* and *C. glabrata*) is observed (Salazar et al. 2020). For that reason, the consumption of probiotic lactobacilli can enhance and restore vaginal homeostasis, thus inhibiting the growth of pathogenic fungi and the formation of fungal biofilms, as it has been widely demonstrated (Wu et al. 2022; Liu et al. 2023). Indeed, several combinations of lactobacilli strains, to be administered either via oral or intravaginal route, are currently under clinical trials (Liu et al. 2023). Furthermore, studies regarding the combination of lactobacilli with lactoferrin (Superti De Seta 2020) and the synergistic effect obtained from the combination of lactobacilli with mannan oligosaccharides (Faustino et al. 2024) demonstrated an improvement in the health of women with vaginal fungal infections and an inhibitory effect on the adhesion of the *C. albicans*, thus highlighting the potential of probiotics and prebiotics as efficient therapeutic candidates for the treatment of fungal infection.

Ultimately, all the abovementioned strategies reported interesting results that represent a significant advancement in the ongoing development of new therapeutic approaches against candidiasis.

### Commercial products

The most common treatments for yeast vaginal infections involve antifungal medications. These can be administered in various forms, including creams, ointments, tablets, and suppositories. Preferable options consist in the use of a cream applied topically in the vaginal area (and used daily for up to 7 days), or a single dose of fluconazole taken orally. Clotrimazole (Lotrimin) and miconazole (Monistat) are widely used and typically effective for mild to moderate infections, as well as oral medication such as fluconazole (Diflucan) (Cleveland Clinic 2022). A list of the top sellers from Amazon is available in Table 2.

**Table 2:** Available commercial products, presentation format, claims, ingredients concentration per serving, treatment duration, and goal

Producer	Product	Presentation	Claims	Ingredients concentration per serving	Treatment duration	Treatment goal
NutraBlast	Boric Life	Vaginal suppositories	Supports odor control; promotes vaginal balance	Boric acid powder (600 mg)	1 for 7 days	Treatment
Lemme	Lemme Purr	Vaginal probiotic gummies	Balanced pH, healthy odor, yeast balance, and flora support + vitamin C for immune health	*Bacillus coagulans* SNZ 1969 (1 billion CFU), pineapple (*Ananas comosus*) powder (100 mg), vitamin C (20 mg)	2 gummies daily	Preventive dietary supplement
Monistat	Monistat 1 combination pack	Vaginal insert + vaginal cream	Cures most vaginal yeast infections; relieves associated external itching and irritation	Miconazole nitrate 1200 mg in vaginal insert and 2% in external cream	External cream – 2 times daily for up to 7 days	Treatment
Love Wellness	Love Wellness – the killer	Vaginal suppositories	Controls vaginal odor and promotes a fresh scent	Boric acid (600 mg)	Use after being intimate, at the end of period, or when experiencing irritation from pH imbalance (for up to 14 days)	Treatment
Azo	Yeast Plus	Oral tablets	Relief from vaginal itching, burning, odor, and discharge	*Candida albicans* and other homeopathic ingredients	1 tablet 3 times a day, as long as symptoms persist	Symptom relief
Amazon	Miconazole 3	Vaginal cream	Yeast infection relief	Miconazole nitrate 4%	3 days	Treatment
Amazon	Tioconazole 1	Ointment	Yeast infection relief	Tioconazole 300 mg (6.5%)	1 dose treatment	Treatment

When infections are resistant to standard treatments, more doses of fluconazole or boric acid capsules (topically administered) may be recommended (CDC 2024 d; Mayo Clinic 2024). Another two drugs that have been recently approved by the FDA for recurrent VVC are ibrexafungerp (Brexafemme) in 2021 and oteseconazole (Vivjoa) in 2022. Only the former was approved in the EU by the European Medicines Agency (EMA).

As previously mentioned, natural treatments (e.g., probiotics, tea tree oil, coconut oil, and garlic) can also be an option for those looking to avoid the side effects of conventional medications or for mild infections. These products are known for their antifungal properties, although the scientific validation for their action against *Candida* infections is often absent. Furthermore, they are generally not as well-studied or as effective as conventional antifungal medications. Nevertheless, and especially for probiotics, they are usually also in the consumers list of preferences, as observed in Table 2.

The global yeast infection treatment market size for VV candidiasis was valued at 4.37 billion in 2023 and is expected to reach USD 7.01 billion by 2031, with a compound annual growth rate (CAGR) of 6.1% during the forecast period of 2024 to 2031. These predictions are based on the rise in autoimmune disorders and yeast infections, as well as market expansion due to the global economic recovery, which enables consumers to access better and higher-quality healthcare (Sabyasachi Ghosh 2023; Data Bridge 2024).

## Conclusion

Vaginal infections caused by *Candida albicans* remain a significant global health concern due to their high recurrence rates and negative impact on quality of life. Understanding the pathogenesis of *C. albicans* infections, particularly its key virulence factors such as biofilm formation, adherence, and phenotypic switching, is crucial for developing more effective prevention and treatment strategies. While antifungal therapies, probiotics, and hygiene practices are widely used, challenges such as antifungal resistance, recurrence, and limited efficacy highlight the need for innovative approaches. Emerging strategies, including novel antifungal agents (e.g., antimicrobial peptides), vaccines, and nanotechnology-based delivery systems (e.g., liposomes), offer promising solutions to improve outcomes and reduce dependency on current treatments.

The immune system plays a critical role in preventing *C. albicans* infections, emphasizing the importance of both innate and adaptive immune responses in restricting fungal colonization and overgrowth. Despite the availability of commercial products such as antifungal creams and/or oral pills and pre-probiotics, long-term efficacy remains limited. Addressing persistent challenges, including patient compliance, access to care, and resistance, requires an interdisciplinary approach that integrates personalized medicine, advanced therapeutic options, and improved healthcare accessibility.

Future research efforts must focus on bridging these gaps to advance prevention and treatment strategies. By fostering a multi-faceted approach that combines scientific innovation with clinical application, significant strides can be made toward reducing the burden of *C. albicans* infections and improving patient quality of life.

## Data Availability

Not applicable.
